# S-Doped Carbon Dot Treatment Alters RNA Processing, Translation, and Protein Degradation Pathways in HeLa Cells

**DOI:** 10.3390/cimb48040349

**Published:** 2026-03-26

**Authors:** Katarina Davalieva, Vanja Ralić, Gjorgji Bozhinovski, Branislava Gemović, Maja D. Nešić, Lela Korićanac, Tanja Dučić, Manuel Algarra, Iva A. Popović, Milutin Stepić, Marijana Petković

**Affiliations:** 1 Research Centre for Genetic Engineering and Biotechnology “Georgi D Efremov”, Macedonian Academy of Sciences and Arts, 1000 Skopje, North Macedonia; katarina@manu.edu.mk (K.D.);; 2 COHERENCE Centre, Department of Atomic Physics, VINČA Institute of Nuclear Sciences, National Institute of the Republic of Serbia, University of Belgrade, 11000 Belgrade, Serbia; vanja.ralic@vin.bg.ac.rs (V.R.); mstepic@vin.bg.ac.rs (M.S.); 3 Department for Bioinformatics and Computational Chemistry, VINČA Institute of Nuclear Sciences, National Institute of the Republic of Serbia, University of Belgrade, 11000 Belgrade, Serbia; gemovic@vin.bg.ac.rs; 4 Department of Molecular Biology and Endocrinology, VINČA Institute of Nuclear Sciences, National Institute of the Republic of Serbia, University of Belgrade, 11000 Belgrade, Serbia; lela@vin.bg.ac.rs; 5 ALBA-CELLS Synchrotron Light Source, Consorcio para la Construcción Equipamiento y Explotación, Del Laboratorio de Luz Sincrotron, 08290 Cerdanyola del Vallés, Spain; 6 INAMAT^2^-Institute for Advanced Materials and Mathematics, Department of Science, Public University of Navarra, 31006 Pamplona, Spain

**Keywords:** S-doped carbon dots, proteomics, bioinformatics, ribosomes, µFTIR

## Abstract

Carbon dots offer excellent physico-chemical properties and biocompatibility for cancer theranostics systems, either as therapeutic agents themselves, or as potential drug carriers. It is, however, postulated that the drug carrier affects the mechanism of action and intracellular target molecules of a drug. Therefore, in the present study, we systematically evaluated protein alterations in HeLa cervical cancer cells after treatment with sulfur-doped carbon dots (S-CDs). Synchrotron Radiation μFTIR spectroscopy and label-free LC–MS/MS proteomics integrated with bioinformatics were used to assess molecular changes. μFTIR revealed a shift and increased intensity of α-helices, indicating structural changes in proteins as a result of the interaction between S-CDs and cells. Proteomic analysis identified 122 statistically significant (*p* ≤ 0.05) proteins with increased abundance and 61 with decreased abundance following S-CD exposure, many of which possess high α-helix content, consistent with μFTIR findings. Functional analyses showed that up-regulated proteins were enriched in molecular adaptor, transporter, and transcription regulator activities, particularly those involved in RNA metabolism and translation. Down-regulated proteins were dominated by protein-modifying enzymes and cytoskeletal components. Pathway enrichment analysis indicated alterations in mRNA processing, ribosomal pathways, translation factors, aminoacyl-tRNA biosynthesis, and proteasome degradation. Key hub proteins included ribosomal proteins and translation initiation factors. S-CD treatment led to opposite regulation of many proteins compared to their regulation in untreated HeLa cells including down-regulation of ribosomal proteins (RPS27L, RPS19, and RPS5), aminoacyl-tRNA biosynthesis proteins (IARS1, LARS1, and MARS1), and proteasome degradation proteins (PSMD2, PSMD3, and PSMD11), which aligns with the observed cytotoxic effect of S-CDs on cervical cancer cells. Overall, these results highlight significant proteomic and structural protein changes induced by S-CDs and support their potential for cervical cancer treatment, warranting further investigation of this nanomaterial’s biological applications.

## 1. Introduction

Carbon dots (CDs), produced from various carbon-rich sources, and by using several approaches [1], are small nanomaterials with a diameter ranging from a few to hundreds of nanometers, possessing suitable optical properties such as fluorescence, as well as biocompatibility for various biomedical applications. The light emission produced by CDs makes them useful for imaging and diagnostic purposes [2], and their interaction with components of chromatin has opened the possibility of their application for detecting a specific cell cycle stage [3,4,5]. CDs also have potential as anti-cancer agents and nanocarriers for chemotherapeutics, for example, in the treatment of glioblastoma or ovarian cancer [6,7]. Doping of CDs with different atoms, including non-metals and metals, represents an option to modify the optical properties of these nanoparticles, as well as to determine the biological target of a drug. Namely, it has been shown that conjugates of CDs with sulfur (S-CDs), as well as with nitrogen (N-CDs), helped overcome the resistance of ovarian cancer cells to cis-Pt, with S-CDs being more effective for this purpose [7]. CDs can also target specific receptors on the surface of the HL-60 leukemia cell line, such as the transferrin receptor, when the protein transferrin was used as a precursor for CD synthesis [8]. These results, along with other observations, led to the hypothesis that a dopant in a nanocarrier can determine the fate of a chemotherapeutic and aid in selecting the target biomolecule.

The interactions between CDs and proteins can result in changes in the protein’s tertiary structure [9], which can have significant implications on their cellular function. At a cellular level, specific changes in secondary structure motifs have been documented in an ovarian cancer cell line following treatment with nanocomposite systems based on CDs [10]. These changes can potentially alter the proteins’ function, leading to novel therapeutic approaches. However, there is a lack of studies that will help illuminate, more broadly, the overall mechanism by which CDs affect biomolecules on a cellular level.

The present lack of comprehensive knowledge about the interaction of nanoparticles with cells, particularly regarding the nanoparticle-induced changes in protein expression in cancer cells, can be overcome by application of state-of-the-art techniques which offer a wide-ranging view into the changes in protein expression. High-resolution Synchrotron Radiation Fourier Transform Infrared Spectroscopy (µFTIR) has been applied to investigate changes in biomolecular structure upon cell interaction with carbon dots or other nanomaterials. This technique has the potential to evaluate changes in the structure of biomolecules at the cellular level and discover cumulative changes resulting from the interaction of a nanoparticle with a cell, triggered by complex signaling pathways [11]. Comparative proteomics, on the other hand, allows identification of proteins with altered expression by simultaneous measurement of hundreds or thousands of molecules and comparison of their abundances between the conditions of interest (for example, treated vs. non-treated, healthy vs. diseased, etc.) in a non-hypothesis-driven way. The power of the comparative proteomics studies is based on the identification of proteome changes without prior biological knowledge or hypothesis and can greatly contribute towards the elucidation of interaction mechanisms of nanoparticles within the cell. This proteomics-based approach, followed by bioinformatics analysis, was successfully implemented in our recent study investigating the effect of the Pd(II) complex on HeLa cells [12].

In our previous work, we demonstrated the potential of S-CDs to overcome the resistance of cancer cells to cis-Pt treatment [7]. In this study, we employed two label-free approaches, LC-MS/MS and µFTIR, to identify overall protein changes in HeLa cells induced by treatment with S-CDs. The high-resolution µFTIR revealed cumulative changes in protein secondary structure after the S-CD treatment, while the comparative proteomics approach identified up- and down-regulated proteins after cell treatment, which, after detailed bioinformatics analysis, pointed to altered functions and cellular pathways. Taken together, the obtained results contribute towards a more comprehensive understanding of the molecular mechanism by which nanoparticles affect cancer cell metabolism.

## 2. Materials and Methods

### 2.1. Reagents for Cell Culture Treatment

Human cervical carcinoma cells (HeLa) were acquired from the American Type Culture Collection (ATCC, Manassas, VA, USA). Poly (sodium-4-styrene sulfonate, PVP), which was used for the synthesis of S-CDs, was purchased from Sigma-Aldrich (Barcelona, Spain). The cell medium, DMEM, was the product of Thermo Fischer Scientific (Waltham, MA, USA). All other chemicals for the cell culture treatment were purchased from Sigma-Aldrich, GmbH (Taufkirchen, Germany).

### 2.2. Synthesis and Characterization of S-CDs

Poly(sodium 4-styrenesulfonate) was used to generate S-CDs following a previously reported synthesis with some modifications [13]. An amount of 100 mg was dissolved in H_2_O (50 mL) and introduced into a Teflon reactor (Savillex Corporation, Eden Prairie, MN, USA), which was then placed in an aluminum reactor to carry out a hydrothermal reaction for 4 h at 200 °C. After cooling down to room temperature, the reaction mixture was purified by filtration (using a 0.20 mm cylindrical filter) and dialyzed (against MilliQ water) overnight. The mixture was subsequently lyophilized to remove all water from the sample. The obtained solid material was fluorescent under the UV light. The morphology of S-CDs was analyzed by a high-resolution Transmission Electron Microscope (HR TEM Thermo Fisher Scientific, Hillsboro, OR, USA) on an FEI Technai F30 operating (Thermo Fisher Scientific, Hillsboro, OR, USA) at an accelerated voltage of 200 kV, equipped with a field emission gun (FEG) and an objective lens SuperTwin. Infrared Spectra (IR) were acquired using a Nicolet iS5 spectroscope (Thermo Fisher Scientific, Madison, WI, USA), and their zeta potential was also determined using a Zetasizer Nano ZS90 (Malvern Instruments Westborough, MA, USA) at 25 °C.

### 2.3. Cell Culture and Viability Assay

HeLa cells were cultured in a DMEM medium at 37 °C and 5% CO_2_. The cytotoxicity of S-CDs (ranging from 1 µg/mL to 100 µg/mL) was measured using a sulforhodamine B (SRB) according to a procedure described elsewhere [14]. The absorbance was measured at 550 nm with a reference wavelength of 690 nm in a microplate reader (Wallac VICTOR2 1420 Multilabel counter, PerkinElmer, Turku, Finland). Two biological replicates were tested in quadruplicate, and Student’s *t*-test was used to analyze the significance of the differences between samples. The differences were significant when the *p*-value was less than 0.05. The results of cell viability were presented as the percentage of the control (mean ± standard deviation).

### 2.4. Preparation of Cells for µ-FTIR Spectroscopy and Registration of Spectra

µ-FTIR spectroscopy was performed on lyophilized cells, which were grown on a round CaF_2_ carrier, 10 mm in diameter and 0.5 mm thick, with a polished window and treated with S-CDs (100 µg/mL) for 24 h. The cell density was 10.000 for control, or 20.000 for treated cells per sample. These concentrations proved optimal for structural change characterization, as they were high enough to affect cell metabolism and low enough to keep most cells alive and available for further analysis. The cell medium was removed, and the cells were washed three times with a sterile physiological solution (0.9% NaCl) and lyophilized. Lyophilization was carried out using a Martin Christ Alpha1–2 freeze-dryer lyophilizer and Labconco Freeze Zone 4.5 LiterFreeze Dryer Systems (Osterode am Harz, Germany) at −50 °C and a vacuum of 0.3 mbar for 24 h.

After the treatment, changes in cell biomolecules were analyzed by µ-FTIR spectroscopy (Synchrotron ALBA, MIRAS beamline, Barcelona, Spain). Synchrotron light as an IR source was coupled to a Vertex 70v spectrometer, 3000 Hyperion microscope, and mercury cadmium telluride detector. The aperture of the FTIR microscope was set to a single-cell size (15 × 15 µm^2^), and 60 cells were analyzed from each group in triplicates. Each single-cell spectrum was acquired by co-adding 256 spectra at a 4 cm^−1^ resolution. All spectra were collected in the 4000–900 cm^−1^ mid-infrared range, and the data acquisition was done by using the OPUS 8.2 (Bruker, Germany) software package. After the acquisition, spectra were analyzed in the protein fingerprint area (1800–1480 cm^−1^), including rubber band baseline correction and vector normalization for every single cell. The second derivative (17 smoothing points, third polynomial order, and vector normalization) was determined for the Amide I protein region (1700–1600 cm^−1^), and the principal component analysis (PCA) for each dataset was performed. All spectral processing and statistical analyses were executed using Orange software (Bioinformatics Laboratory of the University of Ljubljana [15], Version 3.34.0), with the Quasar data analysis package, Version 1.7.0 [16].

### 2.5. Sample Preparation for Proteomics Analysis

For the proteomics analysis, HeLa cells were treated with 100 µg/mL S-CDs and incubated for 48 h. S-CD-treated HeLa cells and controls were prepared in 3 biological replicates each. The cells from each biological replicate were resuspended in 100 μL of Lysis buffer (4% SDS, 5 mM MgCl_2_ × 6H_2_O, 10 mM CHAPS, 100 mM NH_4_HCO_3_, 50 mM DTT) and subsequently lysed by freeze/thaw for a total of 3 times. After sonication for 20 min in an ice bath to further dissolve the proteins and quantification by the Bradford method [17], the samples were stored at −80 °C. Samples were prepared for LC-MS/MS by an in-solution digestion method using RapiGest (Waters Corp., Milford, MA, USA) as previously described in detail [18].

### 2.6. LC-MS/MS Acquisition and Data Processing

Protein profiling was performed on a SYNAPT G2-Si Mass Spectrometer (Waters Corporation) using label-free, data-independent nano-LC-MS/MS acquisition with ion mobility, named UDMS^E^ [19]. The optimal column load for the UDMS^E^ was determined by testing a pool sample (comprising equal amounts of the individual samples), from 50 to 400 ng per run and processing in ProteinLynx Global SERVER (PLGS, version 3.0.3, Waters Corp., Milford, MA, USA). Then, one test run for quality assurance and one run at the determined 200 ng optimal concentration per sample were performed. LC and MS parameters were previously described in detail [20]. Data was searched against the UniProtKB/Swiss-Prot database containing 20,370 proteins (June 2020), with the added sequence of yeast alcohol dehydrogenase (UniProt P00330). Test runs were processed using PLGS (Waters Corp., Milford, MA, USA), while the comparative proteomics analysis was carried out using Progenesis QIP version 4.1 (nonlinear dynamics, Waters Corporation). PLGS low-energy (LE) and high-energy (HE) threshold settings were set to 200 counts and 50 counts, respectively, while the remaining settings as well as the settings of Progenesis QIP were as described previously [12]. The calculated FDR on the whole dataset level was 2.9%.

### 2.7. Bioinformatics Analysis of Proteomics Data

Differentially abundant proteins were selected based on two or more peptide matches per identification and Anova ≤ 0.05. Gene ontology (GO) analysis of the differentially abundant proteins was performed using the Panther Classification System version 19 [21]. For visualization of the enriched GO annotation terms and pathways, we used the Cytoscape (v3.10.4) plug-in ClueGO (v2.0.6) [22] with the following settings: (1) GO term/pathway selection with an interval from 3 to 8 and a minimum of 3 genes (that represent at least 4% of the total number of associated genes) to be associated with a term; (2) pathways with pV ≤ 0.05; (3) GO term/pathway network connectivity (Kappa score) = 0.4; (4) two-sided hypergeometric test; and (5) use of GO term grouping and leading group term based on the highest Kappa score, with min 50% of genes for group merge and min 50% of terms for group merge. For statistically significant enrichments, *p*-values were adjusted for multiple testing using the Bonferroni step-down (Holm–Bonferroni) correction method. STRING analysis [23] was performed to identify protein–protein interaction networks with a high confidence score (0.700), including only query proteins. The Cytoscape plug-in cytoHubba (v0.1) [24] was used for ranking proteins in the network by their network features using the MCC topological analysis method.

## 3. Results

### 3.1. Physico-Chemical and Biological Properties of S-CDs

The size, composition, and surface charge of S-CDs were determined by TEM, measuring zeta potential, and FTIR (Supplementary Document, Figure S1). As shown in Figure S1a,b, the obtained S-CDs had high crystallinity and an average diameter of around 5 nm (*C_f_* scale bar), and the FTIR spectrum (Figure S1c) shows the presence of typical S-containing bonds, around 600–700 cm^−1^ (C-S stretching or C-S-C stretching vibrations), and the presence of S-O vibrations at around 1399, 1118, and 1014 cm^−1^, which are in agreement with the literature data [25]. This indicates that S-CDs are decorated with sulfonyl groups ($-{\mathrm{SO}}_{3}^{-}$), i.e., successfully doping CDs with S-groups. Determination of zeta potential showed that S-CDs are negatively charged with a value of −47 ± 3 mV. The strong negative ζ-potential arises because sulfonyl (${-\mathrm{SO}}_{3}^{-}$) groups are fully ionized in aqueous media, creating a high density of stable negative charges on the nanoparticle surface that generate strong electrostatic repulsion.

S-CDs affected HeLa cell viability, as the highest tested concentration of 100 µg/mL resulted in about 85% viable cells (Supplementary Document, Figure S2), which demonstrates mild toxicity of this nanoparticle. However, our goal was to investigate changes in treated cells which have not led to high toxicity.

### 3.2. SR FTIR Spectroscopical Analysis of Proteins in HeLa Cells

**Figure 1:**
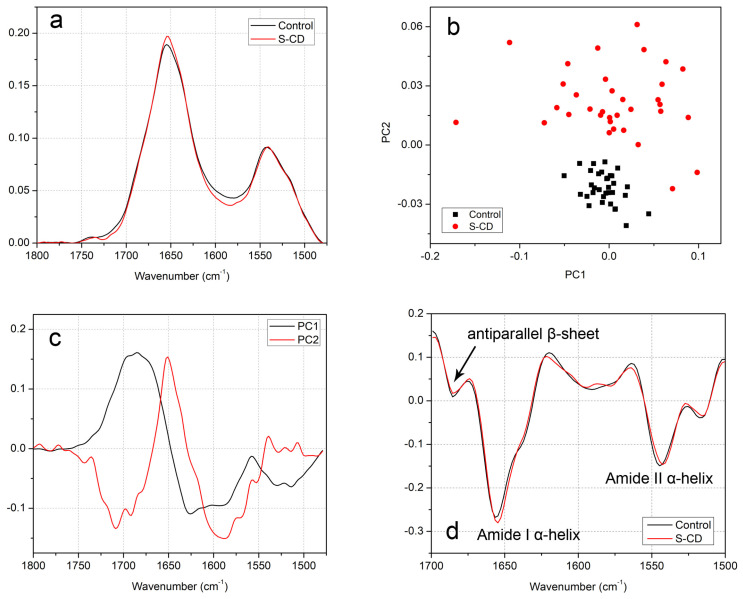
Averaged FTIR spectra of the protein fingerprint region of untreated and S-CD-treated HeLa cells (**a**), PC1 × PC2 scatter plot (**b**), and PC1/PC2 loading plot (**c**). In (**d**), the second derivation of the Amide I and Amide II region is shown, and the arrows indicate secondary structure motifs.

To assess and quantify the cumulative structural and conformational changes in proteins induced by interaction with S-CDs in HeLa cells, we employed µFTIR spectroscopy. Changes in the Amide I and Amide II regions were statistically analyzed using PCA (Figure 1). The protein fingerprint region of the averaged Synchrotron Radiation (SR) FTIR spectra (Figure 1a) spans approximately 1480 to 1800 cm^−1^ and includes the two most prominent protein-associated bands: Amide I (~1655 cm^−1^), corresponding to C=O stretching vibrations, and Amide II (~1543 cm^−1^), associated with N-H bending vibrations [11,26,27]. Both bands reflect the overall protein secondary structure and can be further deconvoluted through second-derivative analysis (Figure 1a,d). Following treatment with S-CDs, the averaged spectra reveal an increased intensity in the Amide I band, while no significant change in the Amide II band is evident at this level. Additionally, after second-derivative processing, a slight shift in the Amide I band is observed from ~1655 cm^−1^ to ~1653 cm^−1^, suggesting interactions between the peptide backbone’s C=O group and functional moieties on the S-CD surface (e.g., ${SO}_{3}^{2-}$ groups).

Although the spectral shifts suggest structural changes in protein α-helices following S-CD treatment, such interpretations require further confirmatory analyses. Nevertheless, the PC1 × PC2 scatter plot (Figure 1b) demonstrates clear group separation, reflecting statistically significant differences between treated and control samples. PCA reveals that 83% of the spectral variance is captured by the first two principal components: PC1 (61%) and PC2 (22%) (Figure 1b,c). Separation of the groups over the PC2 reveals a dominant band at 1651 cm^−1^, typically attributed to α-helical structures, indicating an increased α-helix content in S-CD-treated cells. These PCA-derived findings corroborate the trends observed in the averaged FTIR spectra. These differences in band position and intensity become more apparent upon second-derivative analysis of the spectra. As shown in Figure 1d, a consistent shift in the α-helix-associated peaks is observed in both the Amide I and Amide II regions. This is accompanied by an increase in band intensity, evident in the averaged spectra (Figure 1a). Additionally, a minor shift in the Tyr-associated band (~1515 cm^−1^) suggests potential interactions between S-CDs and tyrosine residues within cellular proteins.

### 3.3. Comparative Proteomics Analysis

**Figure 2:**
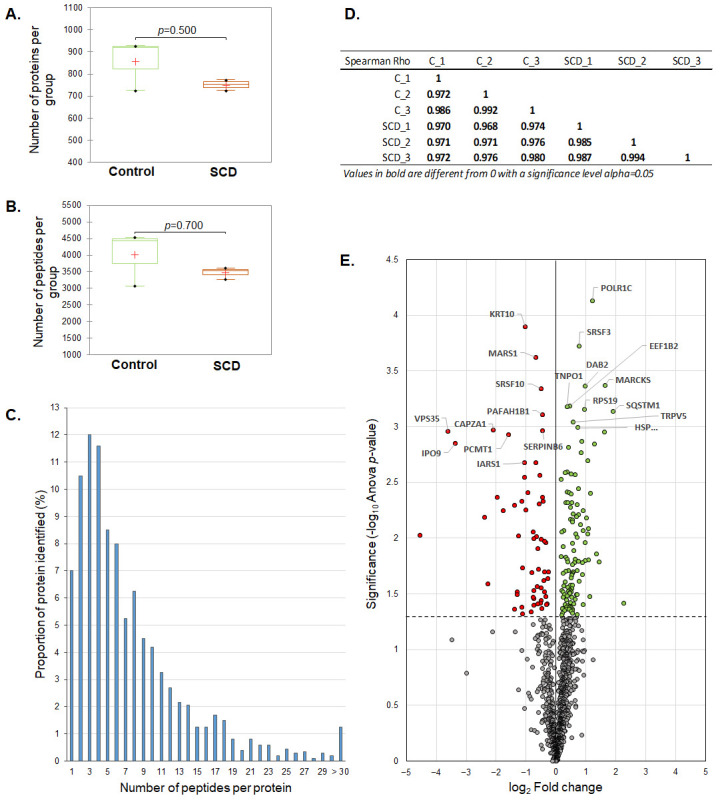
Overview of the proteomics analysis. The numbers of identified proteins (**A**) and peptides (**B**) in S-CD-treated HeLa cells (*n* = 3) and controls (*n* = 3) are represented by box plots. (**C**) Distribution of the number of identified peptides per protein, combined across all runs in the experiment. (**D**) Spearman Rho correlation coefficients between samples for all identified proteins. The correlation matrix is based on normalized protein abundances across the individual samples. (**E**) Volcano plot showing results of differential expression analysis between S-CD-treated HeLa cells and controls. The proteins above the dashed horizontal line represent the 183 statistically significant proteins (Anova ≤ 0.05) where 122 proteins had increased (green) and 61 had decreased (red) abundance in S-CD-treated HeLa cells compared to controls. Top-10 up-regulated and top-10 down-regulated proteins are labeled by gene name.

Comparative proteomics analysis resulted in the identification of 1202 proteins with quantitative values. An average of 3468 and 4010 peptides, corresponding to 751 and 858 proteins in the S-CD-treated and control group, respectively (Figure 2A), were identified by Progenesis QIP. The numbers of peptides and proteins in both groups were comparable (Mann–Whitney test: p_peptides_ = 0.7; p_proteins_ = 0.5) (Figure 2A,B). In total, 93% of the proteins were identified based on two or more peptides (Figure 2C). Furthermore, there was a high and significant correlation (*p* < 0.05) in protein abundance across all samples (Figure 2D), with a median Spearman Rho correlation coefficient of 0.985. The dataset was further filtered to remove reverse sequences, proteins identified on one peptide, and yeast ADH, resulting in a final report that contained 1089 proteins identified on ≥2 peptides (Table S1). Out of these, the statistically significant difference in abundance (Anova ≤ 0.05) between the two groups showed 183 proteins, of which 122 had increased and 61 had decreased abundance in S-CD-treated cells compared with controls (Figure 2E).

### 3.4. Bioinformatics Analysis of Proteomics Data

**Figure 3:**
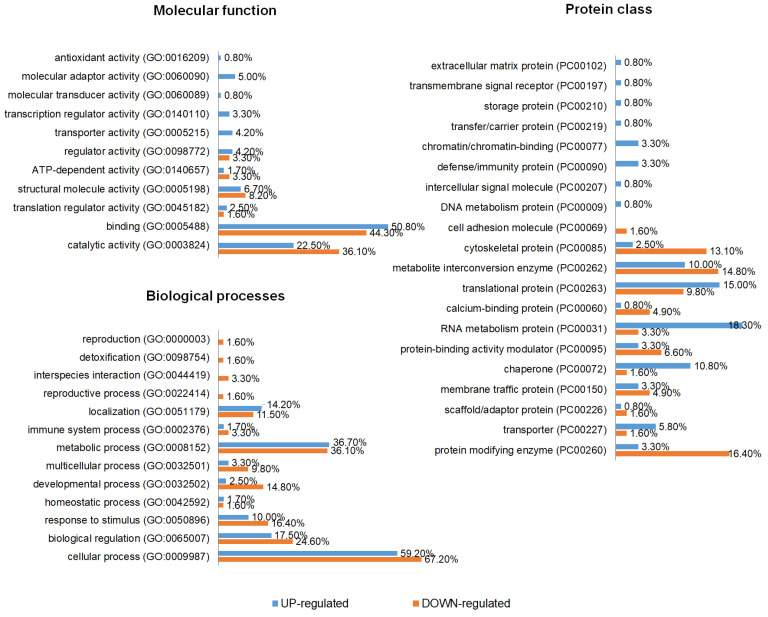
GO annotations of proteins with significantly altered abundance. GO annotations were performed separately for up- and down-regulated proteins. The size of the different functions/processes/classes represents the percentage of involved genes compared to the total number of genes. GO annotations were made according to the Panther Classification System.

To understand the changes in protein abundance in the HeLa cell line treated with S-CDs compared with untreated cells, GO annotations were performed separately for up- and down-regulated proteins. The top-represented molecular functions for both up- and down-regulated proteins were “catalytic activity”, “binding”, and “structural molecule activity”. These categories are commonly enriched in global proteomic datasets and reflect the broad involvement of enzymes and binding proteins in cellular metabolic and regulatory processes. Up-regulated proteins were in addition associated with several other functions such as “molecular adaptor activity”, “transporter activity”, and “transcription regulator activity” (Figure 3), suggesting increased regulation of intracellular signaling and gene expression in response to S-CD treatment. The top-represented biological processes were “cellular process”, “metabolic process”, “biological regulation”, and “response to stimulus”, with no significant difference between up- and down-regulated proteins, which indicates that S-CD exposure affects fundamental cellular functions and triggers cellular adaptation to external stress or treatment. The most-represented protein classes in up-regulated proteins were “RNA metabolism protein”, “translational protein”, “chaperone”, and “transporter”, which suggests an increased demand for protein folding and RNA processing, potentially reflecting cellular efforts to maintain proteostasis under treatment conditions. Up-regulated proteins also contained several unique protein classes, among which the highest-represented were “defense/immunity protein” and “chromatin/chromatin-binding”, indicating possible activation of stress–response pathways and changes in transcriptional regulation. In contrast, the most-represented protein classes in down-regulated proteins were “protein modifying enzyme” and “cytoskeletal protein”, which may suggest alterations in post-translational regulation and cytoskeletal organization following S-CD exposure.

**Figure 4:**
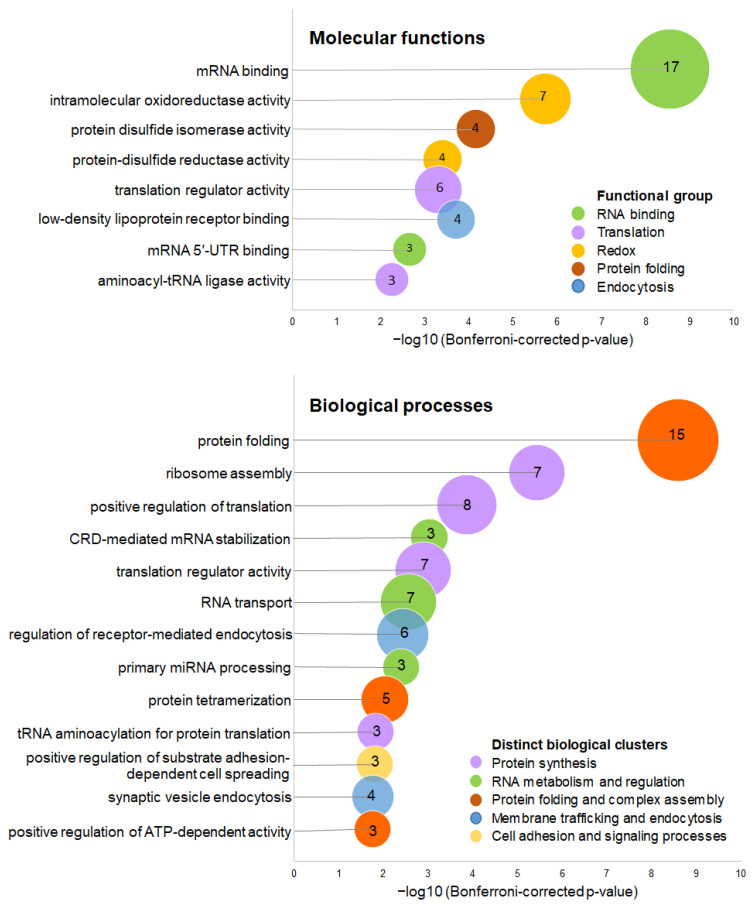
Functional enrichment analysis of up-regulated proteins. GO molecular function and GO biological process enrichment was performed using the ClueGO plug-in in Cytoscape. The *x*-axis represents statistical significance of the enriched functions/processes, expressed as −log10 of the Bonferroni step-down-corrected *p*-value. Bubble size indicates the number of associated genes, while colors represent functional clusters of related molecular functions and biological processes, respectively, as indicated in the graphs’ legends.

Enrichment analysis of the molecular functions and biological processes significantly associated with the differentially abundant proteins was performed using the Cytoscape plug-in ClueGo. Up-regulated differentially abundant proteins were associated with nine molecular functions, clustered in six groups (Figure 4). The molecular function with the highest significance here was “mRNA binding” (*p* = 2.85 × 10^−9^), followed by “intramolecular oxidoreductase activity” (*p* = 1.88 × 10^−6^) and “translation regulator activity, nucleic acid binding” (*p* = 5.08 × 10^−4^). The enrichment of mRNA-binding and translation-regulatory proteins suggests that S-CD treatment may influence post-transcriptional regulation and protein synthesis and is consistent with the observed enrichment of translational proteins in the protein-class analysis and may indicate increased control of mRNA stability and translation efficiency.

A total of 35 biological processes, clustered in 13 groups, were associated with the up-regulated differentially abundant proteins with statistical significance (Figure 4). The top-represented enriched processes with the highest significance were “protein folding” (*p* = 2.58 × 10^−9^), “ribosome assembly” (*p* = 3.72 × 10^−6^), “positive regulation of translation” (*p* = 1.37 × 10^−4^), and “CRD-mediated mRNA stabilization” (*p* = 9.39 × 10^−4^). Together, these processes point to enhanced regulation of the protein synthesis machinery and maintenance of protein quality control. The enrichment of protein-folding pathways is particularly notable, as it may reflect increased cellular stress and the need for chaperone-mediated stabilization of newly synthesized or misfolded proteins. In contrast, down-regulated differentially abundant proteins were associated significantly only with “aminoacyl-tRNA ligase activity” (*p* = 2.81 × 10^−5^) and the biological process of “tRNA aminoacylation for protein translation” (*p* = 5.46 × 10^−4^). These enzymes play a critical role in charging tRNAs during translation. This apparent contrast may indicate a complex regulatory response in which cells adjust different components of the translational machinery to maintain protein synthesis while simultaneously limiting translational capacity under stress conditions.

Pathway enrichment analysis using the KEGG database further supported these observations, identifying ribosome (hsa03010) and aminoacyl-tRNA biosynthesis (hsa00970) as the most significantly enriched pathways (Table 1). Similarly, WikiPathways showed significant association with five pathways, of which the highest significance and coverage had pathways associated with translation (“mRNA processing”, “Cytoplasmic ribosomal proteins”, “Translation factors”) followed by proteasome degradation (“Parkin-ubiquitin proteasomal system pathway”, “Proteasome degradation”). Together, these findings suggest that S-CD treatment strongly affects the cellular protein synthesis machinery. In addition, enrichment of proteasome-related pathways indicates increased protein turnover and degradation and suggests that S-CD treatment may induce proteotoxic stress, leading to activation of cellular quality-control mechanisms that promote chaperone-mediated folding and degradation of misfolded proteins.

**Figure 5:**
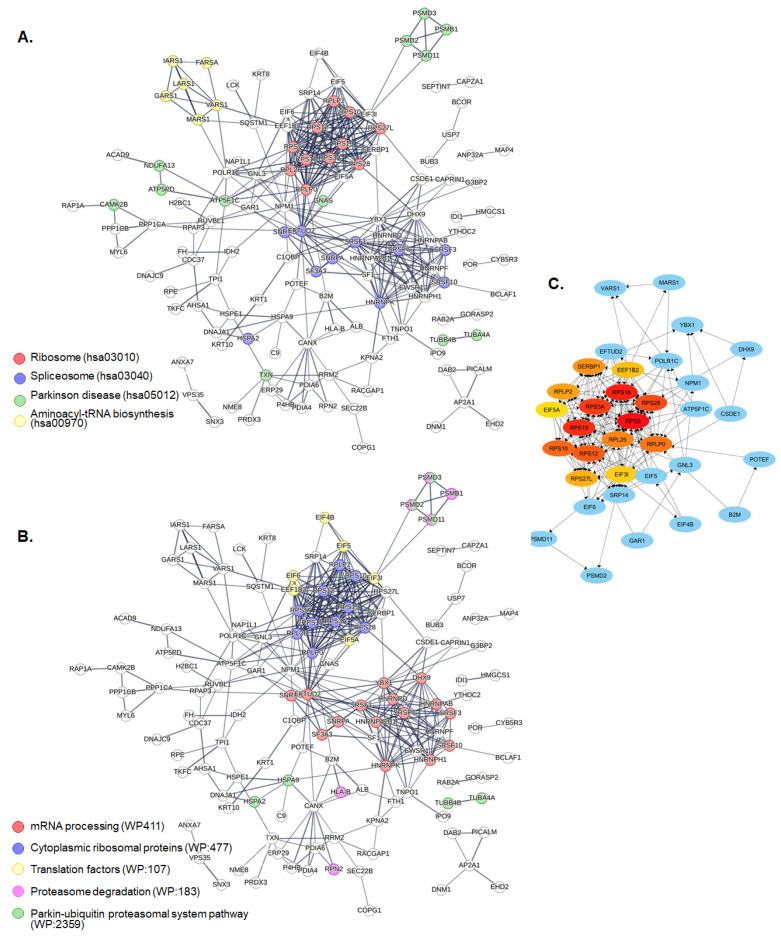
Analysis of the protein–protein interaction network of the differentially abundant proteins. The network was obtained using the STRING database, and proteins associated with significantly enriched pathways according to the (**A**) KEGG and (**B**) WikiPathways databases are colored according to the given legends. The line thickness indicated the strength of data support. (**C**) Cytoscape plug-in cytoHubba analysis results using a STRING-derived protein–protein interaction network. Top-15 essential nodes ranked by MCC scores were highlighted from highly essential (red) to essential (yellow). The graph is extended to include direct linking partners (proteins highlighted with blue).

Protein–protein interaction analysis with STRING indicated that differentially abundant proteins have more interactions among themselves than what would be expected for a random set of proteins of the same size and degree of distribution drawn from the genome (PPI enrichment *p*-value < 1.0 × 10^−16^). Such an enrichment indicates that the proteins are at least partially biologically connected as a group, with at least two well-evident clusters of proteins included in the translation and processing of mRNA (Figure 5A,B). The Cytoscape plug-in CytoHubba was used to explore important nodes in this network. Among the 15 top-ranked proteins, the cytoplasmic ribosomal proteins (RPL26, RPLP0, RPLP2, RPS10, RPS12, RPS18, RPS19, RPS27L, RPS28, RPS3A, RPS5), eukaryotic translation initiation factors (EEF1B2, EIF3I, EIF5A), and ribosome-binding protein SERBP1 had the highest scores, all involved in various stages of protein synthesis (Figure 5C). Together, the PPI network analysis indicates that proteins altered following S-CD treatment are strongly interconnected and mainly associated with pathways regulating mRNA processing, translation, ribosome assembly, and proteasome-mediated protein turnover, highlighting the impact of S-CDs on protein metabolism.

### 3.5. Computational Analysis of Secondary Structures of Differentially Abundant Proteins

The PDB sum database [28] was searched to find the contribution of α-helix and β-sheet structures in the proteins identified as differentially abundant in S-CD-treated cells by comparative proteomics analysis. The results are presented in Table 2, and the contribution to an individual secondary structure of differentially abundant proteins is indicated. The proportion of a particular secondary structure is calculated based on known data, and the results show the number of amino acids which generate this structure. The dominant protein secondary structures in all differentially expressed proteins are α-helices, indicating that the highest proportion of amino acids in these proteins form this secondary structure (Table 2). This further supports the identity of proteins obtained by proteome analysis and confirms the results obtained by µFTIR.

## 4. Discussion

In this work, we tried to unravel the changes in protein metabolism induced by the incubation of HeLa cervical cancer cells with S-CDs. As S-CDs showed small diameters and negative surface charges, these nanoparticles were able to penetrate easily through the cellular membrane and interact with positively charged biomolecules as documented with polystyrene nanoparticles [29].

The use of µFTIR spectroscopy, based on the average spectra of cells and comparative LC-MS/MS analysis, allowed us to map the main S-CD-induced protein changes in cancer cells. µFTIR spectroscopy provides a unique window into cumulative protein structural changes at the single-cell level. Following S-CD treatment, we detected a pronounced increase in α-helical content, most evident in the Amide I band and, to a lesser extent, the Amide II region. This spectral shift reflects a consistent structural trend that aligns with computational biology predictions, which highlight α-helices as the dominant motif among up-regulated proteins. Notably, while proteomics was performed on samples normalized for total protein content, µFTIR captured averaged structural signatures from numerous individual cells. Together, these complementary techniques converge to reveal a clear and coordinated reorganization of protein secondary structure in response to S-CDs, pointing to a broader cellular adaptation at the molecular level. In our previous study, we demonstrated that S-CDs alter protein α-helix structure, both alone and in combination with cisplatin, in adenocarcinoma cells, suggesting these structural changes may contribute to a broader anti-cancer mechanism [7].

To uncover the identity of the dysregulated proteins, we have applied a comparative proteomics analysis coupled with bioinformatics. Of the 183 differentially abundant proteins between S-CD-treated cells and controls, two-thirds showed increased abundance or were up-regulated, while the remaining showed decreased abundance or were down-regulated. Analysis of the molecular functions associated with the dysregulated proteins showed that treatment resulted in an increase in proteins with molecular adaptor activity, transporter activity, and transcription regulator activity. Up-regulated proteins were enriched in RNA metabolism, defense/immunity, and chromatin/chromatin-binding proteins, while the down-regulated proteins were enriched in protein-modifying enzymes and cytoskeletal proteins.

Further, bioinformatic analysis of the proteomics data pointed out several dysregulated cellular pathways. One of the most significantly altered pathways in S-CD-treated HeLa cells according to WikiPathways was mRNA processing. In eukaryotes, this is a crucial step after transcription, where pre-mRNA is modified to become mature mRNA ready for translation, and it involves three main processes: 5′ capping, 3′ polyadenylation, and splicing. These processes ensure stable mRNA that can be transported out of the nucleus and recognized by ribosomes for protein synthesis. The functional link between alterations in mRNA processing, particularly splicing, and cancer is well confirmed by a number of studies [30]. Moreover, overexpression of many spliceosome genes in HeLa cells could play a significant role in the development and progression of cancer [31]. Important for elucidating the effects of S-CDs on HeLa cells are spliceosome proteins that were down-regulated as a result of the treatment. We have detected EFTUD2, SRSF7, and SRSF10 as down-regulated. EFTUD2 represents an important component of the spliceosome, involved in pro-mRNA pruning and splicing. Studies found that EFTUD2 is significantly up-regulated in different tumor tissues and linked to cancer progression and poor prognosis [32]. We have also detected down-regulation of two serine/arginine-rich splicing factors that have multiple key roles in the control of gene expression, including constitutive and alternative pre-mRNA splicing, transcription, mRNA transport, mRNA stability, and translation [33]. A splicing factor SRSF7 has been revealed to play oncogenic roles in multiple cancers [34], while its knockdown inhibited the growth and promoted the apoptosis of colon and lung cancer cells by controlling apoptosis-related splicing events [35]. The splicing factor SRSF10 plays a significant role in cancer development and progression by regulating the alternative splicing of several genes involved in cancer-related pathways. It promotes tumorigenesis in various cancers, including cervical cancer, and its expression is often up-regulated in cancer tissues [36]. The reduced expression of these spliceosome proteins as part of the overall post-transcriptional changes following S-CD exposure may contribute to its potential therapeutic effect against cancer cells and is worth further investigation.

The next significantly altered pathways were cytoplasmic ribosomal proteins (WP:477) or ribosome (hsa03010). Ribosomal proteins play an essential role in the assembly of ribosomes for protein synthesis in the cell [37] and also have multiple extra-ribosomal functions such as in cell cycle progression, genomic stability, regulation of apoptosis, neoplastic transformation, immune signaling, and other functions [38]. Dysregulation of ribosomal proteins can impair cell growth, proliferation, and survival. Deficiency in one ribosomal protein leads to depletion of other ribosomal proteins, and this has deleterious effects on cell survival [38]. Moreover, dysregulation of ribosomal proteins, both as overexpression or reduced expression, is closely correlated with tumorigenesis in various cancers, such as prostate, colorectal, breast, hepatocellular, lung, gastric, and others [39]. Although for some ribosomal proteins, the molecular mechanism explaining this link includes the stabilization and activation of p53 function [40], for most of the so-far-implicated ribosomal proteins in different cancers, the possible mechanism of action remains obscure. Of the eleven differentially expressed ribosomal proteins in our study, ten (RPL26, RPLP2, RPS3A, RPS5, RPS10, RPS12, RPS18, RPS19, RPS27L, RPS28) had increased and only one (RPLP0) had decreased abundance in S-CD-treated HeLa cells compared to controls. Based on the present knowledge about the dysregulated ribosomal proteins in human cancers, the only confirmed link with cervical cancer is the ribosomal protein lateral stalk subunit P0 (RPLP0) [39]. It has been observed that in RPLP0-silenced cells, there is decreased cell viability and cell proliferation, combined with increased cell death [41]. This effect of RPLP0 is achieved through its interaction with Phospholipase A and acyltransferase 4 (PLAAT4), a member of the HREV107 tumor suppressor gene family. Therefore, RPLP0 deficiency directly influences PLAAT4-mediated growth inhibition and cellular apoptosis.

From the overexpressed ribosomal proteins in our study, we detected several that showed an opposite expression trend in S-CD-treated HeLa cells compared to their expression in different cancer cells. These included RPS27L, RPS19, and RPS5, which, based on the expression patterns in different cancers, were postulated to have tumor suppressor activity. Namely, RPS27L was found down-regulated in colon and breast cancer, while its overexpression and high p53 level were demonstrated to enhance DNA repair capacity and trigger apoptosis in cancer cells [42,43]. Low expression of RPS19 was discovered in the feces of CRC patients with bad prognosis, while increased expression is associated with cellular apoptosis through the BAX/p53 pathway [44]. Similarly, down-regulation of RPS5 in colon cancer was associated with tumor progression [45]. The observed alterations in the expression of the above-mentioned ribosomal proteins are in line with the observed cytotoxic effect of S-CDs on cervical cancer cells, supporting the conclusion that the anti-cancer effect of S-CDs might be due to their interaction with the expression of ribosomal proteins.

Another altered pathway resulting from S-CD treatment was eukaryotic translation initiation factors (eIFs) that regulate the translation of mRNA, which is crucial for gene expression. Translation initiation factors play a significant role in cancer development and progression [46]. Dysregulation of these factors, including overexpression, is implicated in the uncontrolled cellular divisions, dysregulated metabolism, and metastasis characteristic of cancer. Many eIFs have been shown to be overexpressed in various cancers, but for some of them, there is an inverse correlation with cancer progression. In our study, we have detected overexpression of several eIFs, including EIF3I, EIF5, and EIF5A, which have established roles as oncogenes, promoting cancer cell growth, invasion, and metastasis [47,48]. However, we have also detected the down-regulation of eukaryotic translation initiation factor 6 (EIF6), which is often found to be overexpressed in tumors, and its role in cancer involves the regulation of ribosome assembly and influencing the translation of specific mRNAs that promote tumorigenesis [49]. However, due to the complex pattern of eIF expression in our study, more research is needed to fully understand the impact of S-CDs on their expression and influence on cancer cell growth.

Aminoacyl-tRNA biosynthesis (hsa00970) is another pathway that was significantly associated with the altered proteins as a result of the impact of S-CDs on HeLa cells. This process is essential for protein synthesis during translation by providing correct attachment of amino acids to their corresponding transfer RNAs (tRNAs), forming aminoacyl-tRNAs. Aminoacyl-tRNA biosynthesis is frequently dysregulated in cancer cells, with altered expression of aminoacyl-tRNA synthetases (ARSs) and tRNAs [50]. This deregulation can promote cancer development and progression by supporting increased protein synthesis demands and contributing to various tumorigenic processes. ARSs were found to be mostly up-regulated in tumors, and their up-regulation often correlated with worse patient survival [51]. However, in our study, half of the proteins included in this pathway, namely IARS1, LARS1, and MARS1, had decreased levels compared to controls. The observed reduction in aminoacyl-tRNA biosynthesis proteins further adds to the potential anti-cancer effect of S-CDs.

Proteasome degradation is a crucial cellular process for protein turnover and quality control, primarily carried out by the 26S proteasome. This process involves tagging proteins with ubiquitin, which signals their degradation by the proteasome into smaller peptides, which are subsequently recycled by the cell [52]. Aberrations in this pathway are commonly observed in many cancers due to the stabilization of oncoproteins or increased degradation of tumor suppressor proteins [53]. As a result of the impact of S-CDs on HeLa cells, we have detected lower levels of several members of the Proteasome 26S Subunit, Non-ATPase (PSMD) gene family, namely PSMD2, PSMD3, and PSMD11. Previous studies have shown that PSMD family genes can play an important role in the progression of tumorigenesis and have been found elevated in various cancers. PSMD1–3 were elevated in breast cancer [54] and PSMD2 was overexpressed in lung cancer [55]. PSMD11 has been identified as a potential biomarker and therapeutic target in several cancers. Elevated levels of PSMD11 were found in different cancers such as lung adenocarcinoma [56], hepatocellular carcinoma [57], and pancreatic cancer [58], and these increased levels were associated with poor prognosis. Since this group of genes is down-regulated in S-CD-treated HeLa cells, we can postulate a potentially therapeutic effect through this group of proteins as well. It is important to note that these proteins, at least those with known secondary structures, are also predominantly organized into α-helices, and the results obtained with µFTIR and computational biology analysis are entirely in line.

## 5. Conclusions

In conclusion, S-CD treatment of HeLa cells resulted in broad dysregulation at the protein level, encompassing fundamental cellular processes that maintain protein levels and cellular function, starting with mRNA processing, through translation, where ribosomal proteins, eukaryotic translation initiation factors, and aminoacyl-tRNA synthetases were included, to the proteasome degradation pathway. Dysregulation of each of these pathways, especially through increased expression of certain components, is a well-established characteristic of cancer. However, S-CD treatment resulted in opposite regulation of many proteins included in these pathways, compared with their regulation in HeLa cells. In particular, the down-regulation of spliceosome proteins and proteasome subunits, together with the reduced abundance of several aminoacyl-tRNA synthetases and the altered expression of ribosomal proteins, suggests that S-CDs interfere with processes essential for cancer cell growth and survival, suggesting a potential anti-cancer effect. Having in mind the mild effect on cell viability, our results imply a high potential of S-CDs, as a part of anti-cervical cancer therapy, potentially as a nanocarrier. The provided insights into the anti-cancer potential of S-CDs represent a solid basis for further functional investigation towards various biological applications of this nanomaterial.

## Figures and Tables

**Table 1:** Pathways associated significantly with the differentially abundant proteins according to KEGG and WikiPathways.

Pathway	*p*-Value *	Coverage (%) **	Associated Proteins
KEGG pathway			
Ribosome (hsa03010)	7.04 × 10^−4^	8.40	RPL26, RPLP0, RPLP2, RPS10, RPS12, RPS18, RPS19, RPS27L, RPS28, RPS3A, RPS5
Parkinson disease (hsa05012)	2.58 × 10^−3^	5.50	ATP5F1C, ATP5PD, CAMK2B, GNAI3, GNAS, NDUFA13, PSMB1, PSMD11, PSMD2, PSMD3, TUBA4A, TUBB4B, TXN
Aminoacyl-tRNA biosynthesis (hsa00970)	5.07 × 10^−3^	13.63	FARSA, GARS1, IARS1, LARS1, MARS1, VARS1
Spliceosome (hsa03040)	4.97 × 10^−2^	7.57	EFTUD2, HNRNPK, SF3A3, SNRPA, SNRPB, SRSF1, SRSF10, SRSF3, SRSF7, HSPA2
WikiPathways			
mRNA processing (WP411)	7.95 × 10^−8^	12.00	DHX9, EFTUD2, HNRNPA2B1, HNRNPAB, HNRNPD, HNRNPH1, HNRNPK, SF3A3, SNRPA, SNRPB, SRSF1, SRSF10, SRSF3, SRSF7, YBX1
Cytoplasmic ribosomal proteins (WP:477)	3.92 × 10^−5^	11.36	RPL26, RPLP0, RPLP2, RPS10, RPS12, RPS18, RPS19, RPS28, RPS3A, RPS5
Parkin-ubiquitin proteasomal system pathway (WP:2359)	2.27 × 10^−3^	9.86	HSPA2, HSPA9, PSMD11, PSMD2, PSMD3, TUBA4A, TUBB4B
Translation factors (WP:107)	3.51 × 10^−3^	12.00	EEF1B2, EIF3I, EIF4B, EIF5, EIF5A, EIF6
Proteasome degradation (WP:183)	8.20 × 10^−3^	9.68	HLA-B, PSMB1, PSMD11, PSMD2, PSMD3, RPN2

* *p*-value corrected with Bonferroni step down; ** percentage of the differentially abundant proteins from this study in the pathway.

**Table 2:** Secondary structure analysis of proteins related to translation/protein synthesis and protein degradation in pathways associated significantly with the differentially abundant proteins according to the KEGG and WikiPathways databases. The table displays only the percentage of α-helices and β-sheets, whereas the complete table, including other structural elements, is provided in the SI, Table S2.

KEGG/WikiPathways	Protein	Part of the Protein with a Known Secondary Structure	Secondary Structure	Contribution of α-Helices and β-Sheets to the Protein Structure
Ribosome (hsa03010) Cytoplasmic ribosomal proteins (WP:477)	RPL26	2–134 (133aa)	3 sheets	24.1%
			5 helices	33.1%
	RPLP0	5–284 (280aa)	3 sheets	11.8%
			12 helices	39.3%
	RPLP2	200–315 (116aa)	4 helices	31.9%
	RPS10	1–99 (99aa)	1 sheet	16.2%
			4 helices	38.4%
	RPS12	10–132 (123aa)	2 sheets	9.8%
			6 helices	44.7%
	RPS18	3–145 (143aa)	1 sheet	5.6%
			9 helices	35.0%
	RPS19	2–145 (144aa)	2 sheets	8.3%
			7 helices	38.9%
	RPS27L	/	/	/
	RPS28	8–68 (61aa)	1 sheet	47.5%
	RPS3A	19–233 (215aa)	1 sheet	32.6%
			6 helices	26.0%
	RPS5	16–204 (189aa)	1 sheet	4.8%
			7 helices	42.3%
Translation factors (WP:107)	EEF1B2	1–91 (91aa)	1 sheet	36.3%
			2 helices	19.8%
	EIF3I	1–312 (312aa)	7 sheets	30.9%
			2 helices	2.0%
	EIF4B	1–81 (81aa)	1 sheet	19.8%
			2 helices	17.3%
	EIF5	1–157 (157aa)	3 sheets	22.3%
			4 helices	24.8%
	EIF5A	15–150 (136aa)	3 sheets	37.5%
			1 helix	5.1%
	EIF6	/	/	/
Parkin-ubiquitin proteasomal system pathway (WP:2359) Proteasome degradation (WP:183)	HSPA2	/	/	/
	HSPA9	46–639 (594aa)	7 sheets	24.4%
			22 helices	30.8%
	PSMD11	1–422 (422aa)	1 sheet	1.7%
			25 helices	59.0%
	PSMD2	1–889 (889aa)	1 sheet	0.9%
			47 helices	50.4%
	PSMD3	18–525 (508aa)	1 sheet	1.2%
			26 helices	54.3%
	TUBA4A	/	/	/
	TUBB4B	1–426 (426aa)	2 sheets	18.1%
			23 helices	41.5%
	HLA-B	1–280 (280aa)	3 sheets	37.9%
			8 helices	23.9%
	PSMB1	29–241 (213aa)	3 sheets	31.5%
			4 helices	31.5%
	RPN2	21–630 (602aa)	6 sheets	22.1%
			20 helices	40.2%

## Data Availability

The original contributions presented in this study are included in the article/Supplementary Material. Further inquiries can be directed to the corresponding authors.

## Supplementary Materials

The following supporting information can be downloaded at https://www.mdpi.com/article/10.3390/cimb48040349/s1.
