# Supplementary material for: S-Doped Carbon Dot Treatment Alters RNA Processing, Translation, and Protein Degradation Pathways in HeLa Cells
Source: Curr Issues Mol Biol. 2026 Mar 26;48(4):349. doi: 10.3390/cimb48040349 (PMC13114559; doi:10.3390/cimb48040349)
Supplement: Supplementary file 1 [file cimb-48-00349-s001.zip › cimb-4178834-supplementary.pdf]

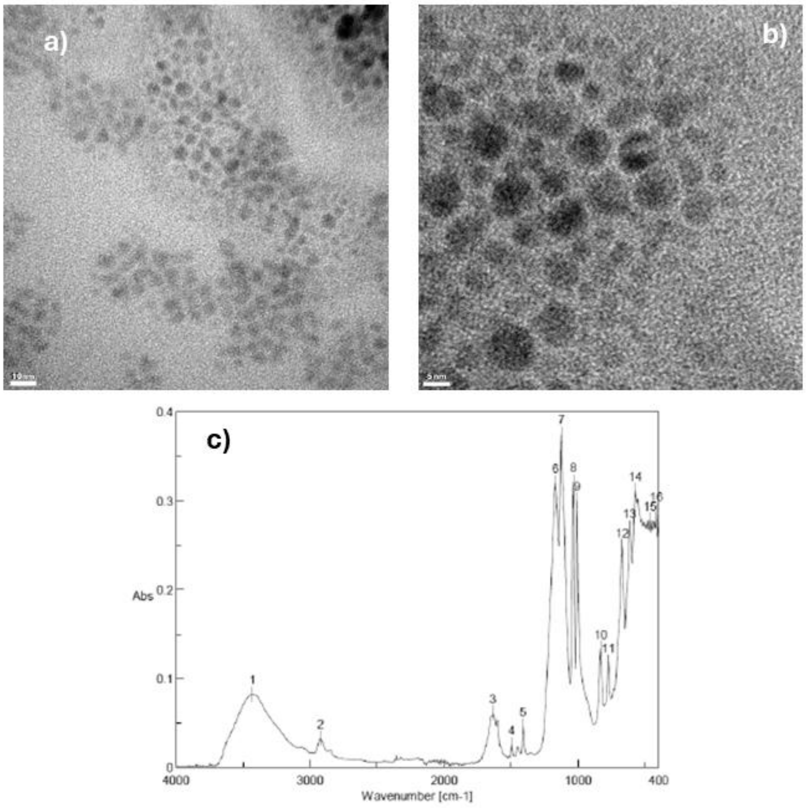

Figure S1. TEM images of S-CDs with lower (a) and higher (b) magnification, and (c) FTIR spectrum of S-CDs.

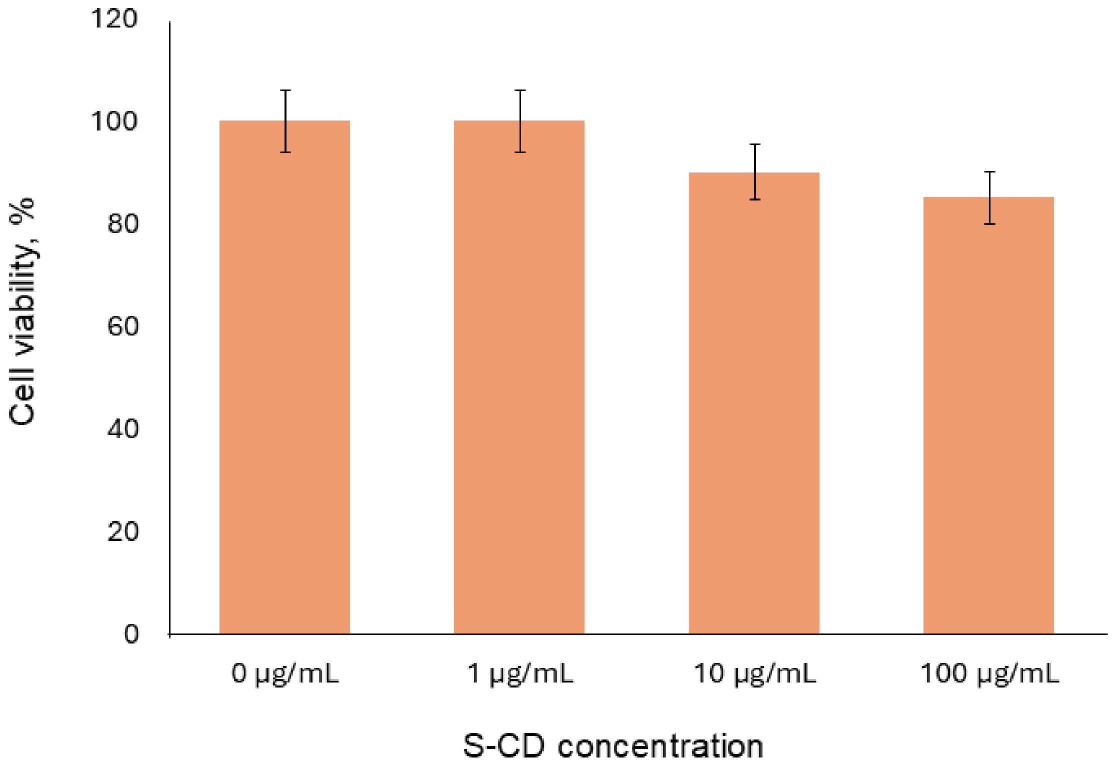

Figure S2. Viability of HeLa cells after the treatment with varying concentrations of S-CD.

Table S1. List of the 183 proteins with differential abundance ($p \leq 0.05$) in S-CD treated HeLa cells and controls obtained by comparative proteomics analysis. Protein identifications were preselected to include only identifications based on ≥2 peptides. For each identified protein, number of total peptides, unique peptides, Anova and fold change are given by Progenesis QIP (Waters corp.).

Accession number	Peptide count	Unique peptides	Description	Anova (p)	Fold change (S-CD treated Controls)
O15160	3	3	DNA-directed RNA polymerases I and III subunit RPAC1 OS=Homo sapiens OX=9606 GN=POLR1C PE=1 SV=1	**7.38E-05**	2.307
P13645	23	17	Keratin_ type I cytoskeletal 10 OS=Homo sapiens OX=9606 GN=KRT10 PE=1 SV=6	**1.27E-04**	-2.038
P84103	6	5	Serine/arginine-rich splicing factor 3 OS=Homo sapiens OX=9606 GN=SRSF3 PE=1 SV=1	**1.89E-04**	1.711
P56192	13	8	Methionine--tRNA ligase_ cytoplasmic OS=Homo sapiens OX=9606 GN=MARS1 PE=1 SV=2	**2.39E-04**	-1.601
P29966	9	8	Myristoylated alanine-rich C-kinase substrate OS=Homo sapiens OX=9606 GN=MARCKS PE=1 SV=4	**4.26E-04**	3.119
P98082	13	11	Disabled homolog 2 OS=Homo sapiens OX=9606 GN=DAB2 PE=1 SV=3	**4.32E-04**	1.954
O75494	5	4	Serine/arginine-rich splicing factor 10 OS=Homo sapiens OX=9606 GN=SRSF10 PE=1 SV=1	**4.54E-04**	-1.408
P24534	8	6	Elongation factor 1-beta OS=Homo sapiens OX=9606 GN=EEF1B2 PE=1 SV=3	**6.55E-04**	1.383
Q92973	6	6	Transportin-1 OS=Homo sapiens OX=9606 GN=TNPO1 PE=1 SV=2	**6.57E-04**	1.294
P39019	5	5	40S ribosomal protein S19 OS=Homo sapiens OX=9606 GN=RPS19 PE=1 SV=2	**7.02E-04**	1.918
Q13501	12	12	Sequestosome-1 OS=Homo sapiens OX=9606 GN=SQSTM1 PE=1 SV=1	**7.26E-04**	3.770
P43034	7	4	Platelet-activating factor acetylhydrolase IB subunit alpha OS=Homo sapiens OX=9606 GN=PAFAH1B1 PE=1 SV=2	**7.76E-04**	-1.373
Q9NQA5	6	4	Transient receptor potential cation channel subfamily V member 5 OS=Homo sapiens OX=9606 GN=TRPV5 PE=1 SV=2	**9.03E-04**	1.496
P48741	9	2	Putative heat shock 70 kDa protein 7 OS=Homo sapiens OX=9606 GN=HSPA7 PE=5 SV=2	**1.01E-03**	1.653
P52907	7	2	F-actin-capping protein subunit alpha-1 OS=Homo sapiens OX=9606 GN=CAPZA1 PE=1 SV=3	**1.07E-03**	-4.268
P35237	2	2	Serpin B6 OS=Homo sapiens OX=9606 GN=SERPINB6 PE=1 SV=3	**1.09E-03**	-1.374
Q96QK1	5	1	Vacuolar protein sorting-associated protein 35 OS=Homo sapiens OX=9606 GN=VPS35 PE=1 SV=2	**1.09E-03**	-12.212
Q96QD8	2	1	Sodium-coupled neutral amino acid transporter 2 OS=Homo sapiens OX=9606 GN=SLC38A2 PE=1 SV=2	**1.12E-03**	3.074

P22061	3	1	Protein-L-isoaspartate(D-aspartate) O-methyltransferase OS=Homo sapiens OX=9606 GN=PCMT1 PE=1 SV=4	**1.17E-03**	-3.013
O60493	5	4	Sorting nexin-3 OS=Homo sapiens OX=9606 GN=SNX3 PE=1 SV=3	**1.36E-03**	1.807
Q96P70	2	1	Importin-9 OS=Homo sapiens OX=9606 GN=IPO9 PE=1 SV=3	**1.40E-03**	-10.351
Q9NYF8	4	2	Bcl-2-associated transcription factor 1 OS=Homo sapiens OX=9606 GN=BCLAF1 PE=1 SV=2	**1.44E-03**	2.417
P07954	7	5	Fumarate hydratase_ mitochondrial OS=Homo sapiens OX=9606 GN=FH PE=1 SV=3	**1.53E-03**	1.338
P67809	9	5	Y-box-binding protein 1 OS=Homo sapiens OX=9606 GN=YBX1 PE=1 SV=3	**1.72E-03**	1.786
Q01581	4	4	Hydroxymethylglutaryl-CoA synthase_ cytoplasmic OS=Homo sapiens OX=9606 GN=HMGCS1 PE=1 SV=2	**2.03E-03**	2.074
P41252	11	6	Isoleucine--tRNA ligase_ cytoplasmic OS=Homo sapiens OX=9606 GN=IARS1 PE=1 SV=2	**2.10E-03**	-2.080
P04264	26	20	Keratin_ type II cytoskeletal 1 OS=Homo sapiens OX=9606 GN=KRT1 PE=1 SV=6	**2.10E-03**	-1.590
P61978	17	16	Heterogeneous nuclear ribonucleoprotein K OS=Homo sapiens OX=9606 GN=HNRNPK PE=1 SV=1	**2.53E-03**	1.298
O43684	5	4	Mitotic checkpoint protein BUB3 OS=Homo sapiens OX=9606 GN=BUB3 PE=1 SV=1	**2.59E-03**	1.232
Q99729	4	3	Heterogeneous nuclear ribonucleoprotein A/B OS=Homo sapiens OX=9606 GN=HNRNPAB PE=1 SV=2	**2.64E-03**	1.419
Q15084	12	10	Protein disulfide-isomerase A6 OS=Homo sapiens OX=9606 GN=PDIA6 PE=1 SV=1	**2.70E-03**	1.549
P35527	17	11	Keratin_ type I cytoskeletal 9 OS=Homo sapiens OX=9606 GN=KRT9 PE=1 SV=3	**2.72E-03**	-1.450
Q9Y678	15	12	Coatomer subunit gamma-1 OS=Homo sapiens OX=9606 GN=COPG1 PE=1 SV=1	**2.85E-03**	-2.074
Q8WXX5	5	4	DnaJ homolog subfamily C member 9 OS=Homo sapiens OX=9606 GN=DNAJC9 PE=1 SV=1	**2.95E-03**	1.124
P14678	3	3	Small nuclear ribonucleoprotein-associated proteins B and B' OS=Homo sapiens OX=9606 GN=SNRPB PE=1 SV=2	**3.60E-03**	1.686
P50502	9	8	Hsc70-interacting protein OS=Homo sapiens OX=9606 GN=ST13 PE=1 SV=2	**3.87E-03**	1.291
Q9H6S0	5	3	3'-5' RNA helicase YTHDC2 OS=Homo sapiens OX=9606 GN=YTHDC2 PE=1 SV=2	**3.90E-03**	1.345
O00231	5	4	26S proteasome non-ATPase regulatory subunit 11 OS=Homo sapiens OX=9606 GN=PSMD11 PE=1 SV=3	**3.92E-03**	-1.913
Q14444	4	2	Caprin-1 OS=Homo sapiens OX=9606 GN=CAPRIN1 PE=1 SV=2	**3.97E-03**	2.233
P25398	7	5	40S ribosomal protein S12 OS=Homo sapiens OX=9606 GN=RPS12 PE=1 SV=3	**4.02E-03**	1.416
Q5CZ79	5	2	Ankyrin repeat domain-containing protein 20B OS=Homo sapiens OX=9606 GN=ANKRD20A8P PE=2 SV=2	**4.27E-03**	-3.881

Q16658	15	12	Fascin OS=Homo sapiens OX=9606 GN=FSCN1 PE=1 SV=3	**4.32E-03**	-1.361
O75534	12	9	Cold shock domain-containing protein E1 OS=Homo sapiens OX=9606 GN=CSDE1 PE=1 SV=2	**4.68E-03**	-2.211
P27348	10	6	14-3-3 protein theta OS=Homo sapiens OX=9606 GN=YWHAQ PE=1 SV=1	**4.69E-03**	-1.354
Q12874	4	4	Splicing factor 3A subunit 3 OS=Homo sapiens OX=9606 GN=SF3A3 PE=1 SV=1	**4.78E-03**	1.263
Q13907	4	3	Isopentenyl-diphosphate Delta-isomerase 1 OS=Homo sapiens OX=9606 GN=IDI1 PE=1 SV=2	**4.80E-03**	1.305
O00273	4	3	DNA fragmentation factor subunit alpha OS=Homo sapiens OX=9606 GN=DFFA PE=1 SV=1	**4.80E-03**	1.434
Q05209	6	5	Tyrosine-protein phosphatase non-receptor type 12 OS=Homo sapiens OX=9606 GN=PTPN12 PE=1 SV=3	**4.91E-03**	-1.476
P55010	4	4	Eukaryotic translation initiation factor 5 OS=Homo sapiens OX=9606 GN=EIF5 PE=1 SV=2	**4.99E-03**	1.632
Q9NNZ3	2	2	DnaJ homolog subfamily C member 4 OS=Homo sapiens OX=9606 GN=DNAJC4 PE=1 SV=1	**5.04E-03**	-2.635
Q9BVP2	5	4	Guanine nucleotide-binding protein-like 3 OS=Homo sapiens OX=9606 GN=GNL3 PE=1 SV=2	**5.30E-03**	1.394
P60660	5	5	Myosin light polypeptide 6 OS=Homo sapiens OX=9606 GN=MYL6 PE=1 SV=2	**5.55E-03**	-2.002
Q9Y265	11	11	RuvB-like 1 OS=Homo sapiens OX=9606 GN=RUVBL1 PE=1 SV=1	**5.66E-03**	-3.380
P09012	3	1	U1 small nuclear ribonucleoprotein A OS=Homo sapiens OX=9606 GN=SNRPA PE=1 SV=3	**5.69E-03**	1.874
P37108	7	6	Signal recognition particle 14 kDa protein OS=Homo sapiens OX=9606 GN=SRP14 PE=1 SV=2	**6.08E-03**	1.475
Q58FF3	6	1	Putative endoplasmin-like protein OS=Homo sapiens OX=9606 GN=HSP90B2P PE=5 SV=1	**6.14E-03**	1.679
P02748	7	6	Complement component C9 OS=Homo sapiens OX=9606 GN=C9 PE=1 SV=2	**6.37E-03**	1.616
P00387	2	1	NADH-cytochrome b5 reductase 3 OS=Homo sapiens OX=9606 GN=CYB5R3 PE=1 SV=3	**6.45E-03**	-5.225
P61769	2	2	Beta-2-microglobulin OS=Homo sapiens OX=9606 GN=B2M PE=1 SV=1	**6.60E-03**	2.019
Q9H1E3	5	5	Nuclear ubiquitous casein and cyclin-dependent kinase substrate 1 OS=Homo sapiens OX=9606 GN=NUCKS1 PE=1 SV=1	**6.74E-03**	1.402
P04844	7	7	Dolichyl-diphosphooligosaccharide--protein glycosyltransferase subunit 2 OS=Homo sapiens OX=9606 GN=RPN2 PE=1 SV=3	**6.85E-03**	1.466
P48735	4	2	Isocitrate dehydrogenase [NADP]_ mitochondrial OS=Homo sapiens OX=9606 GN=IDH2 PE=1 SV=2	**7.18E-03**	1.472

P62714	8	1	Serine/threonine-protein phosphatase 2A catalytic subunit beta isoform OS=Homo sapiens OX=9606 GN=PPP2CB PE=1 SV=1	**7.28E-03**	-201.698
Q8NC51	13	10	Plasminogen activator inhibitor 1 RNA-binding protein OS=Homo sapiens OX=9606 GN=SERBP1 PE=1 SV=2	**7.58E-03**	1.751
Q9UN86	3	3	Ras GTPase-activating protein-binding protein 2 OS=Homo sapiens OX=9606 GN=G3BP2 PE=1 SV=2	**8.16E-03**	2.110
Q9Y240	3	2	C-type lectin domain family 11 member A OS=Homo sapiens OX=9606 GN=CLEC11A PE=1 SV=1	**8.51E-03**	1.874
P31943	12	2	Heterogeneous nuclear ribonucleoprotein H OS=Homo sapiens OX=9606 GN=HNRNPH1 PE=1 SV=4	**8.58E-03**	1.198
P07339	5	4	Cathepsin D OS=Homo sapiens OX=9606 GN=CTSD PE=1 SV=1	**8.76E-03**	-1.701
O95433	8	7	Activator of 90 kDa heat shock protein ATPase homolog 1 OS=Homo sapiens OX=9606 GN=AHSA1 PE=1 SV=1	**8.84E-03**	1.141
Q71UM5	2	1	40S ribosomal protein S27-like OS=Homo sapiens OX=9606 GN=RPS27L PE=1 SV=3	**9.15E-03**	1.575
P63241	5	3	Eukaryotic translation initiation factor 5A-1 OS=Homo sapiens OX=9606 GN=EIF5A PE=1 SV=2	**9.21E-03**	2.096
A5A3E0	13	1	POTE ankyrin domain family member F OS=Homo sapiens OX=9606 GN=POTEF PE=1 SV=2	**9.38E-03**	-23.239
P06239	4	2	Tyrosine-protein kinase Lck OS=Homo sapiens OX=9606 GN=LCK PE=1 SV=6	**9.50E-03**	-2.381
O75131	6	5	Copine-3 OS=Homo sapiens OX=9606 GN=CPNE3 PE=1 SV=1	**9.74E-03**	-1.584
Q96AT9	2	2	Ribulose-phosphate 3-epimerase OS=Homo sapiens OX=9606 GN=RPE PE=1 SV=1	**9.87E-03**	1.619
Q13126	3	2	S-methyl-5'-thioadenosine phosphorylase OS=Homo sapiens OX=9606 GN=MTAP PE=1 SV=2	**1.01E-02**	-1.660
Q9H8Y8	4	3	Golgi reassembly-stacking protein 2 OS=Homo sapiens OX=9606 GN=GORASP2 PE=1 SV=3	**1.02E-02**	-1.416
P56537	5	5	Eukaryotic translation initiation factor 6 OS=Homo sapiens OX=9606 GN=EIF6 PE=1 SV=1	**1.06E-02**	-1.316
P62136	5	2	Serine/threonine-protein phosphatase PP1-alpha catalytic subunit OS=Homo sapiens OX=9606 GN=PPP1CA PE=1 SV=1	**1.09E-02**	-1.272
P62140	4	2	Serine/threonine-protein phosphatase PP1-beta catalytic subunit OS=Homo sapiens OX=9606 GN=PPP1CB PE=1 SV=3	**1.10E-02**	1.964
Q9P0J0	2	2	NADH dehydrogenase [ubiquinone] 1 alpha subcomplex subunit 13 OS=Homo sapiens OX=9606 GN=NDUFA13 PE=1 SV=3	**1.11E-02**	1.461
P01889	5	4	HLA class I histocompatibility antigen_ B alpha chain OS=Homo sapiens OX=9606 GN=HLA-B PE=1 SV=3	**1.18E-02**	1.180

Q3LXA3	6	6	Triokinase/FMN cyclase OS=Homo sapiens OX=9606 GN=TKFC PE=1 SV=2	**1.24E-02**	-1.520
P63092	5	4	Guanine nucleotide-binding protein G(s) subunit alpha isoforms short OS=Homo sapiens OX=9606 GN=GNAS PE=1 SV=1	**1.29E-02**	1.502
P31689	6	2	DnaJ homolog subfamily A member 1 OS=Homo sapiens OX=9606 GN=DNAJA1 PE=1 SV=2	**1.38E-02**	2.540
Q13492	4	3	Phosphatidylinositol-binding clathrin assembly protein OS=Homo sapiens OX=9606 GN=PICALM PE=1 SV=2	**1.46E-02**	1.130
P16435	17	12	NADPH--cytochrome P450 reductase OS=Homo sapiens OX=9606 GN=POR PE=1 SV=2	**1.48E-02**	1.260
Q8N427	4	3	Thioredoxin domain-containing protein 3 OS=Homo sapiens OX=9606 GN=NME8 PE=2 SV=2	**1.54E-02**	1.546
P38646	23	21	Stress-70 protein_ mitochondrial OS=Homo sapiens OX=9606 GN=HSPA9 PE=1 SV=2	**1.55E-02**	1.284
O75947	2	1	ATP synthase subunit d_ mitochondrial OS=Homo sapiens OX=9606 GN=ATP5PD PE=1 SV=3	**1.56E-02**	2.114
P02768	14	13	Albumin OS=Homo sapiens OX=9606 GN=ALB PE=1 SV=2	**1.58E-02**	1.972
P30048	5	5	Thioredoxin-dependent peroxide reductase_ mitochondrial OS=Homo sapiens OX=9606 GN=PRDX3 PE=1 SV=3	**1.60E-02**	1.669
Q15637	6	5	Splicing factor 1 OS=Homo sapiens OX=9606 GN=SF1 PE=1 SV=4	**1.63E-02**	1.305
Q96A08	6	1	Histone H2B type 1-A OS=Homo sapiens OX=9606 GN=H2BC1 PE=1 SV=3	**1.63E-02**	2.718
Q14103	8	5	Heterogeneous nuclear ribonucleoprotein D0 OS=Homo sapiens OX=9606 GN=HNRNPD PE=1 SV=1	**1.65E-02**	1.544
P61604	10	9	10 kDa heat shock protein_ mitochondrial OS=Homo sapiens OX=9606 GN=HSPE1 PE=1 SV=2	**1.65E-02**	1.855
P37802	8	6	Transgelin-2 OS=Homo sapiens OX=9606 GN=TAGLN2 PE=1 SV=3	**1.73E-02**	1.461
Q9UBE0	5	4	SUMO-activating enzyme subunit 1 OS=Homo sapiens OX=9606 GN=SAE1 PE=1 SV=1	**1.86E-02**	-2.169
O43242	4	3	26S proteasome non-ATPase regulatory subunit 3 OS=Homo sapiens OX=9606 GN=PSMD3 PE=1 SV=2	**1.91E-02**	-1.498
P52597	6	3	Heterogeneous nuclear ribonucleoprotein F OS=Homo sapiens OX=9606 GN=HNRNPF PE=1 SV=3	**1.95E-02**	1.619
Q16181	4	3	Septin-7 OS=Homo sapiens OX=9606 GN=SEPTIN7 PE=1 SV=2	**1.95E-02**	1.333
Q6W2J9	5	3	BCL-6 corepressor OS=Homo sapiens OX=9606 GN=BCOR PE=1 SV=1	**1.97E-02**	1.255
P06756	10	7	Integrin alpha-V OS=Homo sapiens OX=9606 GN=ITGAV PE=1 SV=2	**1.99E-02**	-1.308
P06748	12	8	Nucleophosmin OS=Homo sapiens OX=9606 GN=NPM1 PE=1 SV=2	**2.00E-02**	1.489

Q9H0H5	10	7	Rac GTPase-activating protein 1 OS=Homo sapiens OX=9606 GN=RACGAP1 PE=1 SV=1	**2.00E-02**	-1.184
Q9NZN4	3	3	EH domain-containing protein 2 OS=Homo sapiens OX=9606 GN=EHD2 PE=1 SV=2	**2.04E-02**	-1.743
Q13347	6	5	Eukaryotic translation initiation factor 3 subunit I OS=Homo sapiens OX=9606 GN=EIF3I PE=1 SV=1	**2.07E-02**	1.173
P52292	12	10	Importin subunit alpha-1 OS=Homo sapiens OX=9606 GN=KPNA2 PE=1 SV=1	**2.07E-02**	1.454
Q05707	9	5	Collagen alpha-1(XIV) chain OS=Homo sapiens OX=9606 GN=COL14A1 PE=1 SV=3	**2.11E-02**	1.848
Q05193	4	2	Dynamin-1 OS=Homo sapiens OX=9606 GN=DNM1 PE=1 SV=2	**2.13E-02**	1.435
P62269	8	5	40S ribosomal protein S18 OS=Homo sapiens OX=9606 GN=RPS18 PE=1 SV=3	**2.15E-02**	1.444
P42126	3	3	Enoyl-CoA delta isomerase 1_ mitochondrial OS=Homo sapiens OX=9606 GN=ECI1 PE=1 SV=1	**2.27E-02**	1.427
Q9P2J5	23	19	Leucine--tRNA ligase_ cytoplasmic OS=Homo sapiens OX=9606 GN=LARS1 PE=1 SV=2	**2.30E-02**	-1.206
P35080	2	2	Profilin-2 OS=Homo sapiens OX=9606 GN=PFN2 PE=1 SV=3	**2.39E-02**	-1.324
P39687	8	7	Acidic leucine-rich nuclear phosphoprotein 32 family member A OS=Homo sapiens OX=9606 GN=ANP32A PE=1 SV=1	**2.44E-02**	1.697
P55209	6	4	Nucleosome assembly protein 1-like 1 OS=Homo sapiens OX=9606 GN=NAP1L1 PE=1 SV=1	**2.47E-02**	1.236
Q13554	2	1	Calcium/calmodulin-dependent protein kinase type II subunit beta OS=Homo sapiens OX=9606 GN=CAMK2B PE=1 SV=3	**2.57E-02**	-4.838
P10599	5	4	Thioredoxin OS=Homo sapiens OX=9606 GN=TXN PE=1 SV=3	**2.64E-02**	1.398
Q01844	2	2	RNA-binding protein EWS OS=Homo sapiens OX=9606 GN=EWSR1 PE=1 SV=1	**2.64E-02**	1.453
P20618	7	5	Proteasome subunit beta type-1 OS=Homo sapiens OX=9606 GN=PSMB1 PE=1 SV=2	**2.67E-02**	1.188
P11766	4	2	Alcohol dehydrogenase class-3 OS=Homo sapiens OX=9606 GN=ADH5 PE=1 SV=4	**2.72E-02**	-1.560
Q07955	9	7	Serine/arginine-rich splicing factor 1 OS=Homo sapiens OX=9606 GN=SRSF1 PE=1 SV=2	**2.77E-02**	1.306
P68366	11	3	Tubulin alpha-4A chain OS=Homo sapiens OX=9606 GN=TUBA4A PE=1 SV=1	**2.80E-02**	-1.420
P30040	7	6	Endoplasmic reticulum resident protein 29 OS=Homo sapiens OX=9606 GN=ERP29 PE=1 SV=4	**2.84E-02**	1.451
P13667	13	10	Protein disulfide-isomerase A4 OS=Homo sapiens OX=9606 GN=PDIA4 PE=1 SV=2	**2.90E-02**	1.373
Q9UJU6	5	4	Drebrin-like protein OS=Homo sapiens OX=9606 GN=DBNL PE=1 SV=1	**2.95E-02**	1.401
Q9Y3X0	3	2	Coiled-coil domain-containing protein 9 OS=Homo sapiens OX=9606 GN=CCDC9 PE=1 SV=1	**2.96E-02**	-1.678

Q8N392	5	3	Rho GTPase-activating protein 18 OS=Homo sapiens OX=9606 GN=ARHGAP18 PE=1 SV=3	**2.96E-02**	1.148
O95782	9	6	AP-2 complex subunit alpha-1 OS=Homo sapiens OX=9606 GN=AP2A1 PE=1 SV=3	**2.97E-02**	1.334
Q9Y285	3	3	Phenylalanine--tRNA ligase alpha subunit OS=Homo sapiens OX=9606 GN=FARSA PE=1 SV=3	**3.02E-02**	1.187
Q9NZL9	6	6	Methionine adenosyltransferase 2 subunit beta OS=Homo sapiens OX=9606 GN=MAT2B PE=1 SV=1	**3.02E-02**	-1.310
Q9H6T3	3	2	RNA polymerase II-associated protein 3 OS=Homo sapiens OX=9606 GN=RPAP3 PE=1 SV=2	**3.03E-02**	-2.441
P27824	12	11	Calnexin OS=Homo sapiens OX=9606 GN=CANX PE=1 SV=2	**3.05E-02**	1.411
P07237	25	22	Protein disulfide-isomerase OS=Homo sapiens OX=9606 GN=P4HB PE=1 SV=3	**3.08E-02**	1.367
P05387	6	6	60S acidic ribosomal protein P2 OS=Homo sapiens OX=9606 GN=RPLP2 PE=1 SV=1	**3.10E-02**	1.374
Q9NQG5	4	4	Regulation of nuclear pre-mRNA domain-containing protein 1B OS=Homo sapiens OX=9606 GN=RPRD1B PE=1 SV=1	**3.18E-02**	1.610
P62857	3	2	40S ribosomal protein S28 OS=Homo sapiens OX=9606 GN=RPS28 PE=1 SV=1	**3.20E-02**	1.212
P00374	2	1	Dihydrofolate reductase OS=Homo sapiens OX=9606 GN=DHFR PE=1 SV=2	**3.20E-02**	-2.466
Q07021	5	4	Complement component 1 Q subcomponent-binding protein_ mitochondrial OS=Homo sapiens OX=9606 GN=C1QBP PE=1 SV=1	**3.22E-02**	1.623
P02794	5	4	Ferritin heavy chain OS=Homo sapiens OX=9606 GN=FTH1 PE=1 SV=2	**3.30E-02**	1.294
P68371	15	1	Tubulin beta-4B chain OS=Homo sapiens OX=9606 GN=TUBB4B PE=1 SV=1	**3.33E-02**	2.182
P20073	2	1	Annexin A7 OS=Homo sapiens OX=9606 GN=ANXA7 PE=1 SV=3	**3.34E-02**	-1.266
P36405	6	6	ADP-ribosylation factor-like protein 3 OS=Homo sapiens OX=9606 GN=ARL3 PE=1 SV=2	**3.36E-02**	-1.691
P46782	8	7	40S ribosomal protein S5 OS=Homo sapiens OX=9606 GN=RPS5 PE=1 SV=4	**3.37E-02**	1.450
Q08211	21	13	ATP-dependent RNA helicase A OS=Homo sapiens OX=9606 GN=DHX9 PE=1 SV=4	**3.40E-02**	1.173
P08754	6	4	Guanine nucleotide-binding protein G(i) subunit alpha OS=Homo sapiens OX=9606 GN=GNAI3 PE=1 SV=3	**3.48E-02**	-1.678
P54753	4	2	Ephrin type-B receptor 3 OS=Homo sapiens OX=9606 GN=EPHB3 PE=1 SV=2	**3.51E-02**	1.560
Q5TZA2	33	21	Rootletin OS=Homo sapiens OX=9606 GN=CROCC PE=1 SV=1	**3.57E-02**	1.281
Q16543	6	4	Hsp90 co-chaperone Cdc37 OS=Homo sapiens OX=9606 GN=CDC37 PE=1 SV=1	**3.61E-02**	-1.416
P61019	4	4	Ras-related protein Rab-2A OS=Homo sapiens OX=9606 GN=RAB2A PE=1 SV=1	**3.77E-02**	1.387
Q9BTE7	3	1	DCN1-like protein 5 OS=Homo sapiens OX=9606 GN=DCUN1D5 PE=1 SV=1	**3.81E-02**	4.768

P51451	3	2	Tyrosine-protein kinase Blk OS=Homo sapiens OX=9606 GN=BLK PE=1 SV=3	**3.85E-02**	-1.440
P08133	17	17	Annexin A6 OS=Homo sapiens OX=9606 GN=ANXA6 PE=1 SV=3	**3.90E-02**	-1.519
Q13200	4	4	26S proteasome non-ATPase regulatory subunit 2 OS=Homo sapiens OX=9606 GN=PSMD2 PE=1 SV=3	**3.90E-02**	-1.231
Q9BWM7	9	7	Sideroflexin-3 OS=Homo sapiens OX=9606 GN=SFXN3 PE=1 SV=3	**3.91E-02**	1.511
P41250	13	11	Glycine--tRNA ligase OS=Homo sapiens OX=9606 GN=GARS1 PE=1 SV=3	**3.91E-02**	1.234
P05388	10	9	60S acidic ribosomal protein P0 OS=Homo sapiens OX=9606 GN=RPLP0 PE=1 SV=1	**3.96E-02**	-1.251
Q2NL68	2	2	Proline and serine-rich protein 3 OS=Homo sapiens OX=9606 GN=PROSER3 PE=1 SV=1	**3.96E-02**	1.876
Q9HAW9	2	2	UDP-glucuronosyltransferase 1A8 OS=Homo sapiens OX=9606 GN=UGT1A8 PE=1 SV=1	**3.98E-02**	-1.662
P61247	12	11	40S ribosomal protein S3a OS=Homo sapiens OX=9606 GN=RPS3A PE=1 SV=2	**4.05E-02**	1.304
P05787	24	14	Keratin_ type II cytoskeletal 8 OS=Homo sapiens OX=9606 GN=KRT8 PE=1 SV=7	**4.10E-02**	1.384
P60174	20	16	Triosephosphate isomerase OS=Homo sapiens OX=9606 GN=TPI1 PE=1 SV=4	**4.15E-02**	1.282
P46783	4	3	40S ribosomal protein S10 OS=Homo sapiens OX=9606 GN=RPS10 PE=1 SV=1	**4.15E-02**	1.379
Q9UBK8	4	2	Methionine synthase reductase OS=Homo sapiens OX=9606 GN=MTRR PE=1 SV=4	**4.18E-02**	-2.223
Q9NY12	2	2	H/ACA ribonucleoprotein complex subunit 1 OS=Homo sapiens OX=9606 GN=GAR1 PE=1 SV=1	**4.20E-02**	1.418
P31350	3	3	Ribonucleoside-diphosphate reductase subunit M2 OS=Homo sapiens OX=9606 GN=RRM2 PE=1 SV=1	**4.25E-02**	1.180
P61254	4	3	60S ribosomal protein L26 OS=Homo sapiens OX=9606 GN=RPL26 PE=1 SV=1	**4.26E-02**	1.166
O95747	9	9	Serine/threonine-protein kinase OSR1 OS=Homo sapiens OX=9606 GN=OXSR1 PE=1 SV=1	**4.29E-02**	1.166
Q15029	18	11	116 kDa U5 small nuclear ribonucleoprotein component OS=Homo sapiens OX=9606 GN=EFTUD2 PE=1 SV=1	**4.29E-02**	-1.395
A6NMY6	14	2	Putative annexin A2-like protein OS=Homo sapiens OX=9606 GN=ANXA2P2 PE=5 SV=2	**4.33E-02**	1.374
P23588	16	13	Eukaryotic translation initiation factor 4B OS=Homo sapiens OX=9606 GN=EIF4B PE=1 SV=2	**4.33E-02**	1.458
P27816	15	12	Microtubule-associated protein 4 OS=Homo sapiens OX=9606 GN=MAP4 PE=1 SV=3	**4.36E-02**	1.279
Q93009	4	3	Ubiquitin carboxyl-terminal hydrolase 7 OS=Homo sapiens OX=9606 GN=USP7 PE=1 SV=2	**4.36E-02**	-2.621
P14923	3	3	Junction plakoglobin OS=Homo sapiens OX=9606 GN=JUP PE=1 SV=3	**4.36E-02**	1.361
P26640	6	4	Valine--tRNA ligase OS=Homo sapiens OX=9606 GN=VARS1 PE=1 SV=4	**4.43E-02**	1.470

Q9H845	3	3	Complex I assembly factor ACAD9_ mitochondrial OS=Homo sapiens OX=9606 GN=ACAD9 PE=1 SV=1	**4.43E-02**	1.213
P22626	11	7	Heterogeneous nuclear ribonucleoproteins A2/B1 OS=Homo sapiens OX=9606 GN=HNRNPA2B1 PE=1 SV=2	**4.52E-02**	1.410
Q16629	5	3	Serine/arginine-rich splicing factor 7 OS=Homo sapiens OX=9606 GN=SRSF7 PE=1 SV=1	**4.60E-02**	-1.769
P54652	21	3	Heat shock-related 70 kDa protein 2 OS=Homo sapiens OX=9606 GN=HSPA2 PE=1 SV=1	**4.72E-02**	1.467
P36542	3	2	ATP synthase subunit gamma_ mitochondrial OS=Homo sapiens OX=9606 GN=ATP5F1C PE=1 SV=1	**4.72E-02**	1.167
P62834	2	2	Ras-related protein Rap-1A OS=Homo sapiens OX=9606 GN=RAP1A PE=1 SV=1	**4.76E-02**	-2.163
P61106	4	2	Ras-related protein Rab-14 OS=Homo sapiens OX=9606 GN=RAB14 PE=1 SV=4	**4.86E-02**	1.640
O75396	4	4	Vesicle-trafficking protein SEC22b OS=Homo sapiens OX=9606 GN=SEC22B PE=1 SV=4	**4.94E-02**	1.145

Table S2. Secondary structure analysis of proteins identified as related to translation/protein synthesis and protein degradation in pathways associated significantly with the differentially abundant proteins according to KEGG and WikiPathways databases

KEGG/WikiPathways pathway	Protein	Part of the Protein with a Known Secondary Structure	Secondary Structure	Contribution of α-Helices and β-Sheets to the Protein Structure
Ribosome (hsa03010)/ Cytoplasmic ribosomal proteins (WP:477)	RPL26	2-134 (133aa)	**3 sheets**<br>8 strands<br>**5 helices**<br>3 helix-helix interacs, 11 beta turns, 1 gamma turn	**24.1%**<br><br>**33.1%**
	RPLP0	5-284 (280aa)	**3 sheets**<br>9 strands<br>**12 helices**<br>17 helix-helix interacs, 28 beta turns, 4 gamma turns	**11.8%**<br><br>**39.3%**
	RPLP2	200-315 (116aa)	**4 helices**<br>10 helix-helix interacs, 7 beta turns, 9 gamma turns	**31.9%**
	RPS10	1-99 (99aa)	**1 sheet**<br>3 strands<br>**4 helices**<br>5 helix-helix interacs, 7 beta turns, 2 gamma turns	**16.2%**<br><br>**38.4%**
	RPS12	10-132 (123aa)	**2 sheets**<br>4 strands<br>**6 helices**<br>8 helix-helix interacs, 8 beta turns	**9.8%**<br><br>**44.7%**
	RPS18	3-145 (143aa)	**1 sheet**<br>2 strands<br>**9 helices**<br>6 helix-helix interacs, 14 beta turns	**5.6%**<br><br>**35.0%**
	RPS19	2-145 (144aa)	**2 sheets**<br>4 strands<br>**7 helices**<br>9 helix-helix interacs, 16 beta turns, 2 gamma turns	**8.3%**<br><br>**38.9%**
	RPS27L	/	/	/

	RPS28	8-68 (61aa)	**1 sheet**<br>4 strands<br>4 beta turns	**47.5%**
	RPS3A	19-233 (215aa)	**1 sheet**<br>8 strands<br>**6 helices**<br>4 helix-helix interacs, 21 beta turns, 3 gamma turns	**32.6%**<br><br>**26.0%**
	RPS5	16-204 (189aa)	**1 sheet**<br>2 strands<br>**7 helices**<br>13 helix-helix interacs, 22 beta turns, 2 gamma turns	**4.8%**<br><br>**42.3%**
Translation factors (WP:107)	EEF1B2	1-91 (91aa)	**1 sheet**<br>4 strands<br>**2 helices**<br>1 helix-helix interac, 14 beta turns, 3 gamma turns	**36.3%**<br><br>**19.8%**
	EIF3I	1-312 (312aa)	**7 sheets**<br>23 strands<br>**2 helices**<br>30 beta turns, 1 gamma turn	**30.9%**<br><br>**2.0%**
	EIF4B	1-81 (81aa)	**1 sheet**<br>4 strands<br>**2 helices**<br>9 beta turns, 5 gamma turns	**19.8%**<br><br>**17.3%**
	EIF5	1-157 (157aa)	**3 sheets**<br>9 strands<br>**4 helices**<br>5 helix-helix interacs, 15 beta turns	**22.3%**<br><br>**24.8%**
	EIF5A	15-150 (136aa)	**3 sheets**<br>10 strands<br>**1 helix**<br>16 beta turns, 3 gamma turns, 1 disulphide	**37.5%**<br><br>**5.1%**
	EIF6	/	/	/
Parkin-ubiquitin proteasomal system pathway (WP:2359)	HSPA2	/	/	/
	HSPA9	46-639 (594aa)	**7 sheets**<br>26 strands<br>**22 helices**	**24.4%**<br><br>**30.8%**

			21 helix-helix interacs, 46 beta turns, 11 gamma turns	
	PSMD11	1-422 (422aa)	**1 sheet**<br>3 strands<br>**25 helices**<br>48 helix-helix interacs, 55 beta turns, 4 gamma turns	**1.7%**<br><br>**59.0%**
	PSMD2	1-889 (889aa)	**1 sheet**<br>2 strands<br>**47 helices**<br>37 helix-helix interacs, 114 beta turns, 13 gamma turns	**0.9%**<br><br>**50.4%**
	PSMD3	18-525 (508aa)	**1 sheet**<br>3 strands<br>**26 helices**<br>31 helix-helix interacs, 69 beta turns, 5 gamma turns	**1.2%**<br><br>**54.3%**
	TUBA4A	/	/	/
	TUBB4B	1-426 (426aa)	**2 sheets**<br>13 strands<br>**23 helices**<br>22 helix-helix interacs, 23 beta turns, 3 gamma turns	**18.1%**<br><br>**41.5%**
Proteasome degradation (WP:183)	HLA-B	1-280 (280aa)	**3 sheets**<br>17 strands<br>**8 helices**<br>8 helix-helix interacs, 18 beta turns, 2 disulphides	**37.9%**<br><br>**23.9%**
	PSMB1	29-241 (213aa)	**3 sheets**<br>12 strands<br>**4 helices**<br>11 helix-helix interacs, 13 beta turns, 2 gamma turns	**31.5%**<br><br>**31.5%**
	PSMD11	1-422 (422a)	**1 sheet**<br>3 strands<br>**25 helices**<br>48 helix-helix interacs, 55 beta turns, 4 gamma turns	**1.7%**<br><br>**59.0%**
	PSMD2	1-889 (889aa)	**1 sheet**	**0.9%**

			2 strands<br>**47 helices**<br>37 helix-helix interacs, 114 beta turns, 13 gamma turns	 **50.4%**
	PSMD3	18-525 (508aa)	**1 sheet**<br>3 strands<br>**26 helices**<br>31 helix-helix interacs, 69 beta turns, 5 gamma turns	**1.2%**<br><br>**54.3%**
	RPN2	21-630 (602aa)	**6 sheets**<br>22 strands<br>**20 helices**<br>34 helix-helix interacs, 37 beta turns, 11 gamma turns, 1 disulphide	**22.1%**<br><br>**40.2%**
